# Beyond Aluminum Loading: How Aluminum Coordination Controls Acidity and Catalytic Performance of Al-SBA-16 in CO_2_‑to-DME Conversion

**DOI:** 10.1021/acsami.5c18904

**Published:** 2025-12-29

**Authors:** Fausto Secci, Valentina Mameli, Patrícia A. Russo, Paula Soares-Santos, Luciano Atzori, Mauro Mureddu, Nicola Pinna, João Rocha, Carla Cannas

**Affiliations:** † Department of Chemical and Geological Sciences, 3111 University of Cagliari, S.S.554 bivio per Sestu, Monserrato 09042 , CA, Italy; ‡ Consorzio Interuniversitario Nazionale per la Scienza e Tecnologia dei Materiali (INSTM), Via Giuseppe Giusti 9, Firenze 50121, FI, Italy; § Department of Chemistry and The Center for the Science of Materials Berlin, 9373 Humboldt-Universität zu Berlin, Berlin 12489, Germany; ∥ CICECO − Aveiro Institute of Materials, Department of Chemistry, University of Aveiro, Aveiro 3810-193, Portugal; ⊥ Sotacarbo S.p.A., Grande Miniera di Serbariu, Carbonia 09013 , SU, Italy

**Keywords:** mesostructured aluminosilicates, SS-NMR, acid sites, dimethyl ether, catalysts

## Abstract

Mesostructured aluminosilicates (Al-SBA-16) with Si/Al molar ratios of 10, 15, and 20 were synthesized and evaluated as methanol dehydration catalysts for the one-pot conversion of CO_2_ to dimethyl ether (DME). Increasing the Al content was expected to enhance activity by generating additional acid sites. Although catalytic tests confirmed higher DME selectivity at lower Si/Al (higher Al content), the gain was modest relative to the nominal increase in Al amount, motivating a closer examination of Al incorporation and its contribution to Bro̷nsted acidity. To address this, ^27^Al and ^29^Si solid-state NMR were combined with pyridine adsorption FT-IR. ^27^Al NMR resolved framework tetrahedral Al alongside extra-framework penta- and octa-coordinated species. Higher Si/Al ratios favored framework incorporation, whereas increased Al loading promoted segregation as amorphous Al_2_O_3_. ^29^Si MAS/CP-MAS supported partial framework substitution (subtle Q^4^ shift) together with a slight increase in Q^3^/Q^2^ (silanol/Si–O–Al) contributions. FTIR corroborated these findings, showing only a moderate increase in the amount of Bro̷nsted sites with decreasing Si/Al and a greater persistence of Lewis sites at high temperature. This work demonstrates that catalytic performance in CO_2_-to-DME conversion is controlled not only by the nominal Al content of Al-SBA-16 but also by the coordination and distribution of Al species between framework and extra-framework environments, establishing a direct structure–acidity–activity relationship that guides the design of more efficient aluminosilicate catalysts.

## Graphical abstract

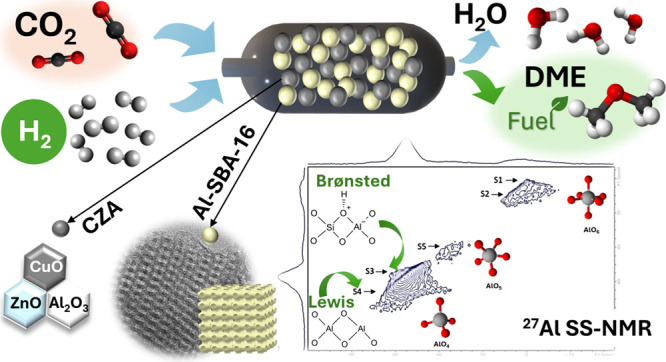

## Introduction

One of the most concerning global challenges is the rapid increase in the CO_2_ concentration in the atmosphere, primarily driven by energy production, industrial activities, and transportation. As the main cause of anthropogenic global warming, CO_2_ emissions are linked to severe climate impacts, such as ice melting, desertification, and sea level rise. To mitigate these effects, carbon capture and utilization (CCU) strategies have gained significant attention. CCU involves capturing CO_2_ from industrial exhaust streams and converting it into valuable chemicals and fuels through hydrogenation.^1–8^ The resulting products, known as electrofuels (e-fuels), are synthesized using hydrogen generated via water electrolysis powered by renewable energy, thereby offering a sustainable pathway to store intermittent renewable energy. Among e-fuels, dimethyl ether (DME) stands out as a particularly promising candidate.^9–16^ DME can be directly used in diesel engines as a fuel or additive, while its oxygenated nature ensures cleaner combustion, reducing particulate and VOC emissions and eliminating SO_*x*_ and NO_*x*_ pollutants.

The conversion of CO_2_ to DME proceeds through two sequential reactions. First, CO_2_ is hydrogenated to methanol:^17–25^

$${\mathrm{CO}}_{2}+3H_{2}⇌{\mathrm{CH}}_{3}\mathrm{OH}+H_{2}O$$

This reaction is typically catalyzed by Cu-based systems promoted with ZnO and stabilized by a third oxide phase, such as Al_2_O_3_ or ZrO_2_.^26–31^ The methanol formed is then dehydrated to DME according to

$$2{\mathrm{CH}}_{3}\mathrm{OH}⇌{\mathrm{CH}}_{3}{\mathrm{OCH}}_{3}+H_{2}O$$

Methanol dehydration is generally carried out over solid acidic catalysts, notably γ-Al_2_O_3_ and zeolites, similar to those used in the syngas-to-DME process.^32–46^ γ-Al_2_O_3_ is widely used due to its low cost, high surface area, thermal stability, and presence of Lewis acid sites of weak and moderate strength. However, its catalytic performance declines over time because of Lewis sites deactivation.^35,36,46–48^ The problem is particularly acute in the CO_2_-to-DME conversion, where water is produced not only during methanol dehydration but also via CO_2_ hydrogenation and the reverse water-gas shift (RWGS) reaction. Water competes for adsorption on the γ-Al_2_O_3_ Lewis acid sites, thereby reducing catalyst activity.

Zeolites exhibit greater resistance to water adsorption than γ-Al_2_O_3_,^13,35,46,49^ attributed to their Bro̷nsted acid sites and a lower surface affinity for water, especially in high Si/Al ratio materials. However, while strong Bro̷nsted sites are highly active for methanol dehydration, they can also promote the formation of undesirable byproducts.^50–53^ Beyond acidity, the structural features of zeolites, such as pore channels, cage dimensions, and crystallite size, strongly influence coke formation and catalyst stability. Large cages connected by narrow pores hinder the diffusion of intermediates and byproducts, favoring their accumulation and subsequent transformation into carbonaceous deposits.^32,54^

Catalytic performance is further influenced by crystallite size: nanocrystalline zeolites enhance methanol conversion, DME selectivity, and reduce coke deposition compared to microcrystalline analogues, owing to enhanced mass transfer.^44^ Among zeolite frameworks, ferrierite generally outperforms over ZSM-5 in both nano- and microcrystalline forms.^44,55,56^ The introduction of mesoporosity provides an additional advantage, facilitating diffusion and limiting coke buildup. This beneficial effect has been observed in hierarchical zeolites as well as ZSM-5/MCM-41 composites.^57,58^

Building on our previous work,^47^ where three mesostructured aluminosilicates with identical Si/Al ratios (15) but different pore arrangements were compared with mesostructured γ-Al_2_O_3_ as methanol dehydration catalysts, the aluminosilicates consistently outperformed γ-Al_2_O_3_ due to the presence of Bro̷nsted acid sites. Among them, Al-SBA-16 was found to be the most promising material, offering a high surface density of active sites.

Despite its promising characteristics, Al-SBA-16 remains relatively underexplored compared to other mesostructured aluminosilicates such as Al-SBA-15 and Al-MCM-41, as well as conventional acidic catalysts such as zeolites and γ-Al_2_O_3_. In particular, detailed investigations correlating the local coordination environment of aluminum species with the resulting acidity and catalytic performance are not common in scientific literature.

Motivated by these gaps, this study investigates three Al-SBA-16 samples with varying Si/Al ratios (10, 15, and 20) to elucidate how aluminum content affects their structural, textural, morphological, and acidic properties. A particular emphasis is placed on establishing a clear correlation among aluminum coordination, the type and strength of acid sites, and the resulting catalytic performance, a relationship that, to date, remains insufficiently addressed in the literature. The ultimate goal is to identify the optimal composition and gain fundamental insight into the structure–acidity–activity interplay governing methanol dehydration on Al-SBA-16 catalysts.

## Experimental Section

### Chemicals

The chemicals used for the syntheses were the following: PEG20-PPG70-PEG20 (Pluronic P-123) average Mn ∼5800 (Aldrich Chemistry), tetraethyl orthosilicate (TEOS) 98% (Aldrich Chemistry), aluminum chloride hexahydrate (AlCl_3_·6H_2_O) 99% (Alfa Aesar), and absolute ethanol (CH_3_CH_2_OH) (Honeywell Fluka), commercial Cu-based redox catalyst (CZA, provided by Alfa Aesar), and alpha-alumina (≥98%, Sigma-Aldrich).

### Synthesis of Al-SBA-16

Al-SBA-16 was prepared according to the procedure reported in ref.^47^ Typically, to obtain 1.5 g of product, 2.36 g of Pluronic P-123 were dissolved in 45 mL of absolute ethanol under continuous stirring at room temperature (RT). After 2 h, 5.34 g of tetraethyl orthosilicate (TEOS), 0.285–0.570 g of AlCl_3_·6H_2_O (depending on the desired Si/Al ratio), and 0.5 mL of water were added to the solution. The mixture was continuously stirred for 24 h and then transferred into a 21 cm-diameter Petri dish, which was placed in a controlled-humidity chamber (40% RH, 25 °C). After 2 days of aging, the obtained gel was calcined at 600 °C for 5 h (heating rate: 2 °C min^–1^) to obtain the final Al-SBA-16 material.

### Characterization Techniques

Small-angle XRD patterns (SA-XRD, 2θ = 0.7–6°) were acquired on a Seifert X3000 diffractometer with a θ–θ geometry, equipped with a Cu anode (Kα radiation of 1.5418 Å). The lattice parameter of the mesostructure was calculated using the formula $a_{0}=d_{110}\sqrt{2}$ .^59,60^ Wide-angle XRD patterns (WA-XRD, 2θ = 10–80°) were recorded on a PANalytical X′ pert Pro (Malvern PANalytical, Malvern, UK) equipped with a copper X-ray source.

The textural properties were determined by nitrogen adsorption–desorption isotherms at −196 °C, measured using a Micromeritics ASAP 2020 instrument. All samples were pretreated under vacuum at 250 °C (heating ramp, 1 °C/min) for 12 h. The Brunauer–Emmett–Teller (BET) specific surface area (SA) was calculated from the adsorption data in the 0.05–0.25 *P*/*P*_0_ range. The total pore volume (*V*_p_) was calculated at *P*/*P* ^0^ = 0.9975, and the Barrett–Joyner–Halenda (BJH) model was applied to the adsorption branch isotherm to determine the mean pore diameter (*D*_p_); the pore size distribution width values associated with BJH measurements were calculated by fitting the BJH curves with a Gaussian function. The pore wall thickness (*T*_w_) was calculated with the formula $T_{w}=a_{0}\frac{\sqrt{3}}{2}-D_{p}$ .^61,62^

The samples were prepared for inductively coupled plasma-optical emission spectrometry (ICP-OES) analysis using an alkaline fusion procedure. Particularly, 100 mg of a finely ground sample, previously heated at 120 °C overnight, was mixed with 1.5 g of lithium tetraborate (Li_2_B_4_O_7_) in a 10 mL platinum crucible and then put into a muffle oven preheated at 1000 °C for 1 h. The content of the crucible was then completely recovered by dissolving it in 100 mL of 5 M HNO_3_. The obtained solution was then diluted with Milli-Q water into a 250 mL volumetric flask to obtain a 2 M HNO_3_ solution for ICP analysis; finally, the solution was filtered with a 0.45 μm syringe filter. ICP-OES analyses were carried out using an Agilent 5110 device (Agilent, Santa Clara, CA, USA). The calibration line was performed using eight standard solutions with a concentration range of 1–150 mg L^–1^ for Al and 1.5–200 mg L^–1^ for Si. The concentration of each element was determined as the mean value of all of the available wavelengths. Each sample was analyzed three times.

A JEOL JEM 1400-PLUS transmission electron microscope operating at a voltage of 120 kV and a field emission gun FEI TALOS F200S 200 kV microscope were used to acquire the transmission electron microscopy (TEM) micrographs. Elemental composition was determined by energy-dispersive X-ray spectroscopy (EDX). For TEM analysis, fine powders were first dispersed in ethanol and sonicated, and the obtained suspensions were deposited onto 200 mesh carbon-coated copper grids.

Thermogravimetric analysis (TGA) was carried out using a PerkinElmer STA 6000 (Waltham, MA, USA) in the 25–900 °C temperature range, with a heating ramp of 10 °C/min under a 40 mL/min O_2_ flow.

The total surface acidity of the catalysts was evaluated by temperature-programmed NH_3_ desorption analyses (NH_3_-TPD), carried out using a TPD/R/O 1100 apparatus (Thermo Fisher Scientific, Waltham, MA, USA). Prior to the analysis, 75 mg of sample was pretreated at 550 °C for 1 h under a 30 mL/min N_2_ flow. Then, pulses of pure ammonia (0.5 mL) were admitted to the sample at 100 °C using He as the gas carrier (50 mL/min); the adsorption was considered complete when the area of the ammonia pulses remained constant. Finally, the NH_3_-TPD analysis was performed under a continuous He flow (50 mL/min) by increasing the temperature from 100 to 600 °C (heating rate, 10 °C/min), followed by an isothermal period of 30 min.

The nature of the acid sites was investigated by pyridine adsorption Fourier-transform infrared spectroscopy (Py-FT-IR)**.** Spectra were acquired on a Nicolet iS50 spectrometer (Thermo Fischer Scientific) equipped with a custom glass cell allowing the sample to be switched between heating and measurement positions. The cell was evacuated (*<*1.3 × 10^–3^ Pa) by using a rotative pump and a turbomolecular pump. Further details on the experimental setup were provided in a previous work.^63^ The FT-IR spectra were acquired in the range 1700–1400 cm^–1^. Before the analysis, each sample was prepared to form a 13 mm self-supported pellet; particularly, 15–20 mg of the sample was pressed for 2–3 min at 2500–3000 kg by using a hydraulic press. The obtained pellet was subsequently introduced into the cell to undergo a thermal treatment at 250 °C (heating ramp of 7.5 °C/min) for 1 h under a high vacuum in order to allow a complete desorption of water. The sample, maintained under high vacuum, was then put in the measurement position and cooled down to room temperature, and its FT-IR spectrum was recorded as a background. Pyridine vapor was then sent into the cell, allowing it to reach a pressure of about 267 Pa with pyridine vapor and maintaining these conditions for 10 min. The cell containing the sample was again evacuated at RT, and the FTIR spectrum was acquired. The spectrum acquisition was repeated after heating the sample at different temperatures (100, 200, and 300 °C) under high vacuum; after each treatment, the sample was cooled to room temperature before recording the spectrum. The thermal treatments at increasing temperatures allowed us to monitor the progressive desorption of pyridine from the acid sites. The areas of the IR bands associated with each type of site were integrated to quantify the number of acid sites occupied by pyridine at each temperature. The band at about 1455 cm^–1^ was used to determine the number of Lewis acid sites, using an integrated molar extinction coefficient (IMEC) of 2.22 cm/μmol, whereas the band at about 1545 cm^–1^ was used to calculate the number of Bro̷nsted acid sites, with an IMEC of 1.67 cm/μmol.^64^

^29^Si magic-angle spinning (MAS) NMR spectra were recorded on a Bruker Avance III 400 (9.4 T) wide-bore spectrometer at 79.5 MHz, using a 7 mm probe with a 5 kHz spinning rate. Spectra were acquired with a 40° pulse flip angle and a 60 s recycle delay. Chemical shifts are quoted in parts per million from TMS with Q_8_M_8_ used as an external reference (low-frequency peak set to −109.68 ppm). For ^29^Si cross-polarization (CP) MAS experiments, ^1^H and ^29^Si 90° pulses were 3.25 and 5.23 ms, respectively. The CP step was implemented with a contact time of 8 ms, a 50–100% ramp on the ^1^H channel. The recycle delay was 5s.

^27^Al (MAS) NMR spectra were recorded on a Bruker Avance III, 700 MHz (16.4 T) narrow-bore spectrometer at 182.4 MHz. Chemical shifts were referenced to 1 M Al­(NO_3_)_3_ aqueous solution (0 ppm). Single-quantum spectra were recorded on a 1.9 mm probe with a spinning rate of 35 kHz using single-pulse excitation with a pulse length of 0.24 μs (10° flip angle), and a 1 s recycle delay. Triple-quantum (3QMAS) experiments were recorded on a 4 mm probe, under MAS at 15 kHz using a standard z-filter three-pulse sequence. Pulse lengths were 3.25 μs for the excitation pulse, 1.3 μs for the conversion pulse, and 6 μs for the z-filter π/2 pulse. A recycle delay of 1 s was used, with isotropic projections obtained after shearing transformation.

### Catalytic Tests

A customized Microactivity Effi (PID Eng&Tech) bench-scale plant, equipped with a high-pressure fixed-bed stainless steel reactor (length 304.8 mm, inner diameter 9.1 mm), was employed for the DME production catalytic tests. The catalytic bed was held inside the isothermal region of the reactor by a porous plate (made of Hastelloy C, 20 μm) and quartz wool. The dehydration catalysts were tested by physically mixing them with a commercial Cu-based redox catalyst (CZA). Both CZA and the acidic catalysts were first ground separately in an agate mortar to obtain fine powders. Subsequently, 50 mg of CZA, 200 mg of acidic catalyst, and 3.2 g of α-Al_2_O_3_ (used as an inert diluent) were physically mixed with a steel spatula in a Teflon weighing boat. An overall bed volume of ca. 3 cm^3^ was thus obtained and, as a result, under a constant inlet flow rate, the gas hourly space velocity (GHSV) was 48,000 N cm^3^ g_cat_^–1^ h^–1^. First, all fresh catalysts were reduced in situ under a H_2_/N_2_ gas mixture (H_2_, 15 vol % in N_2_) at 250 °C for 2 h under atmospheric pressure. Then, at the same temperature, a gaseous stream made up of a 3:1 (molar ratio) mixture of H_2_ and CO_2_ and 10 vol % of N_2_ (internal standard for gas chromatographic analysis) was fed, and the pressure was allowed to reach 3.0 MPa. Feed mixture preparation (consisting of a certified gas cylinder mixture) is carried out with a dedicated mass flow controller: Bronkhorst “Mini Cori Flow” with an accuracy of ± 0.2%. The pressure control is based on a high-speed precision servo-controlled valve with an accuracy of ±0.1 bar. Once the system reached the steady state in 1 h on stream, analyses were performed repeatedly on the reaction stream during the run and conducted for 24 h. The analyses were performed with a 7890B (Agilent) gas chromatograph featuring a flame ionized detector (FID) and a thermal conductivity detector (TCD) for carbon-based compounds and permanent gases, respectively. The components of the outlet gas mixture were separated by two columns connected in series: CO_2_, CH_3_OH, DME, CH_3_CH_3_, and CH_3_CH_2_CH_3_ were separated by an HP-PLOT Q (Agilent) column (length of 30 m, inner diameter of 0.53 mm, and film thickness of 40 μm), while an HP-PLOT Molesieve (Agilent) column (length of 30 m, inner diameter of 0.53 mm, and film thickness of 50 μm) was used for the separation of H_2_, N_2_, CH_4_, and CO. To avoid product condensation, the pipelines connecting the plant gas outlet to the gas chromatograph inlet were heated at 180 °C. The CO_2_ conversion $\left({X_{\mathrm{CO}}}_{2}\right)$ and the products selectivity (SP, with P: CH_3_OH, DME, or CO) were calculated according to ref.^65^ using the following equations:

XCO2=[XCO2,inXN2,in−XCO2,outXN2,outXCO2,inXN2,in]×100%

Sn=[(nXN2)outXCO2,inXN2,in−XCO2,outXN2,out]×100%

The calculated uncertainty of the experimental flow rates together with that related to the gas chromatographic reading of CO_2_, CH_3_OH, CH_3_OCH_3_, and CO gives a value not exceeding 2%.

## Results and Discussion

Small-angle X-ray diffraction (SA-XRD) patterns of all three samples (Figure 1a) show characteristic reflections of the cubic SBA-16 mesostructure (Im3m), with a main (110) peak at 1.2° and a secondary (200) peak at about 1.4°. Variation in the Si/Al ratio did not induce any significant shift in the diffraction peaks, indicating that the mean pore diameter of the mesostructure remained unchanged. The 2θ values of the 110 reflection were used to calculate the cell parameter (*a*_0_) of the mesostructure, and the results (Table 1) revealed no remarkable differences among the samples. However, Al-SBA-16_20 displayed sharper diffraction peaks compared with those of the other aluminosilicates, indicating a higher degree of mesopore ordering, likely favored by its more siliceous framework.

**Figure 1:**
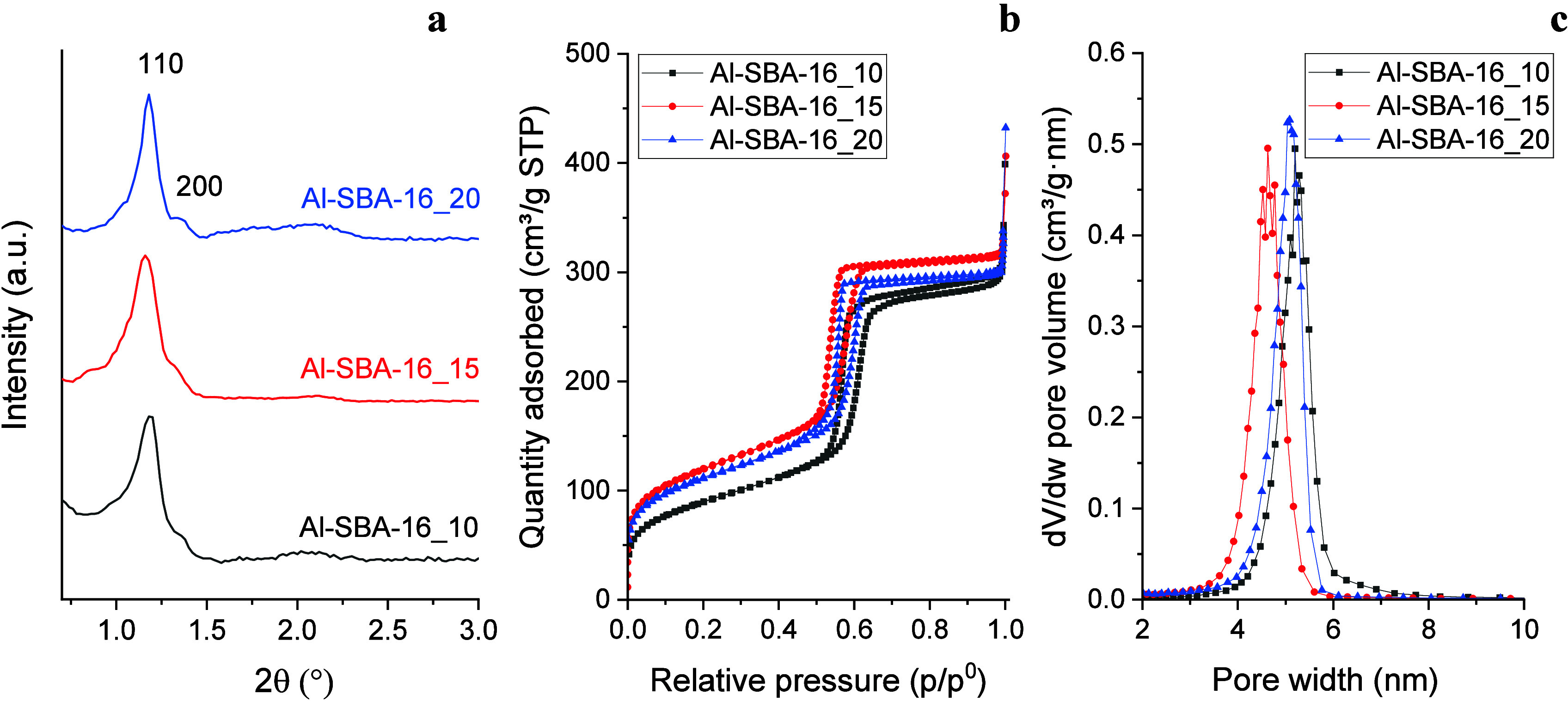
SA-XRD patterns (a), nitrogen physisorption isotherms (b), and BJH adsorption pore size distribution (c) of the Al-SBA-16 samples with different Si/Al ratios (i.e., 10, 15, and 20).

**Table 1:** BET Surface Area (SA), Pore Volume (*V*_p_), Cell Parameter (*a*_0_), Mean BJH Pore Diameter (*D*_p_) with Associated Pore Size Distribution Width, Wall Thickness (*T*_w_), Nominal Si/Al Ratio (Si/Al_nom_), Si/Al Ratio with Standard Deviation Determined by ICP-OES (Si/Al_ICP_), and Si/Al Ratio with Standard Deviation Determined by EDX (Si/Al_EDX_) of the Al-SBA-16 Samples with Different Si/Al Ratios (i.e., 10, 15, and 20)

	SA (m^2^/g)	*V*_p_ (cm^3^/g)	*a*_0_ (nm)	*D*_p_ (nm)	*T*_w_ (nm)	Si/Al_nom_	Si/Al_ICP_	Si/Al_EDX_
Al-SBA-16_10	313	0.53	10.6	5.2 (0.6)	4.0	10	9.9 (0.6)	6.9 (3.6)
Al-SBA-16_15	412	0.49	10.7	4.6 (0.6)	4.7	15	15.0 (0.9)	14.1 (1.6)
Al-SBA-16_20	381	0.47	10.6	5.1 (0.6)	4.1	20	18.6 (1.1)	17.5 (0.14)

Wide-angle X-ray diffraction (WA-XRD) points out that all Al-SBA-16 samples have an amorphous nature (Figure S1a), only showing a broad band at about 23°, attributed to amorphous silica.

All samples present nitrogen physisorption isotherms characteristic of mesoporous materials (type IVa), with a steep capillary condensation branch and a hysteresis loop of type H2, typical of 3D pore networks with large cavities interconnected by smaller pores, as in the cubic SBA-16 structure^59,61,66^ (Figure 1b). For all samples, capillary condensation occurred at relative pressures of 0.5–0.6, indicating comparable mean pore diameters. BJH analysis confirmed mean pore sizes of 5 nm, with Al-SBA-16_15 showing a slightly smaller value (4.6 nm) than Al-SBA-16_10 and Al-SBA-16_20 (5.1–5.2 nm) (Figures 1c and S2a; Table 1). All samples displayed narrow pore size distributions (±0.6 nm).

Surface area values (Table 1) were similar for Al-SBA-16_15 (412 m^2^/g) and Al-SBA-16_20 (381 m^2^/g), while Al-SBA-16_10 showed a 22% lower surface area (313 m^2^/g). In contrast, pore volumes were comparable across all samples (0.47–0.53 cm^3^/g).

The actual mean Si/Al ratios were determined by ICP-OES to allow for accurate quantification and comparison with the nominal synthesis values. The outcomes are summarized in Table 1. The Si/Al ratios measured by ICP-OES closely matched the nominal values, as expected, given the absence of a washing step in the synthesis.

TEM imaging (Figure 2) showed the presence of ordered mesopore structures in all three samples, with interconnected channels arranged in the cubic symmetry typical of SBA-16. Determination of the pore size from TEM images yielded mean values of 5.0 (±0.5) nm for Al-SBA-16_10, 4.7 (±0.5) nm for Al-SBA-16_15, and 4.9 (±0.5) nm for Al-SBA-16_20. Although pore size determinations by TEM have intrinsic limitations, these values are in very good agreement with the mean pore sizes and distributions obtained from BJH analysis (Table 1), again showing the slightly smaller pores of Al-SBA-16_15 compared with the other samples.

**Figure 2:**
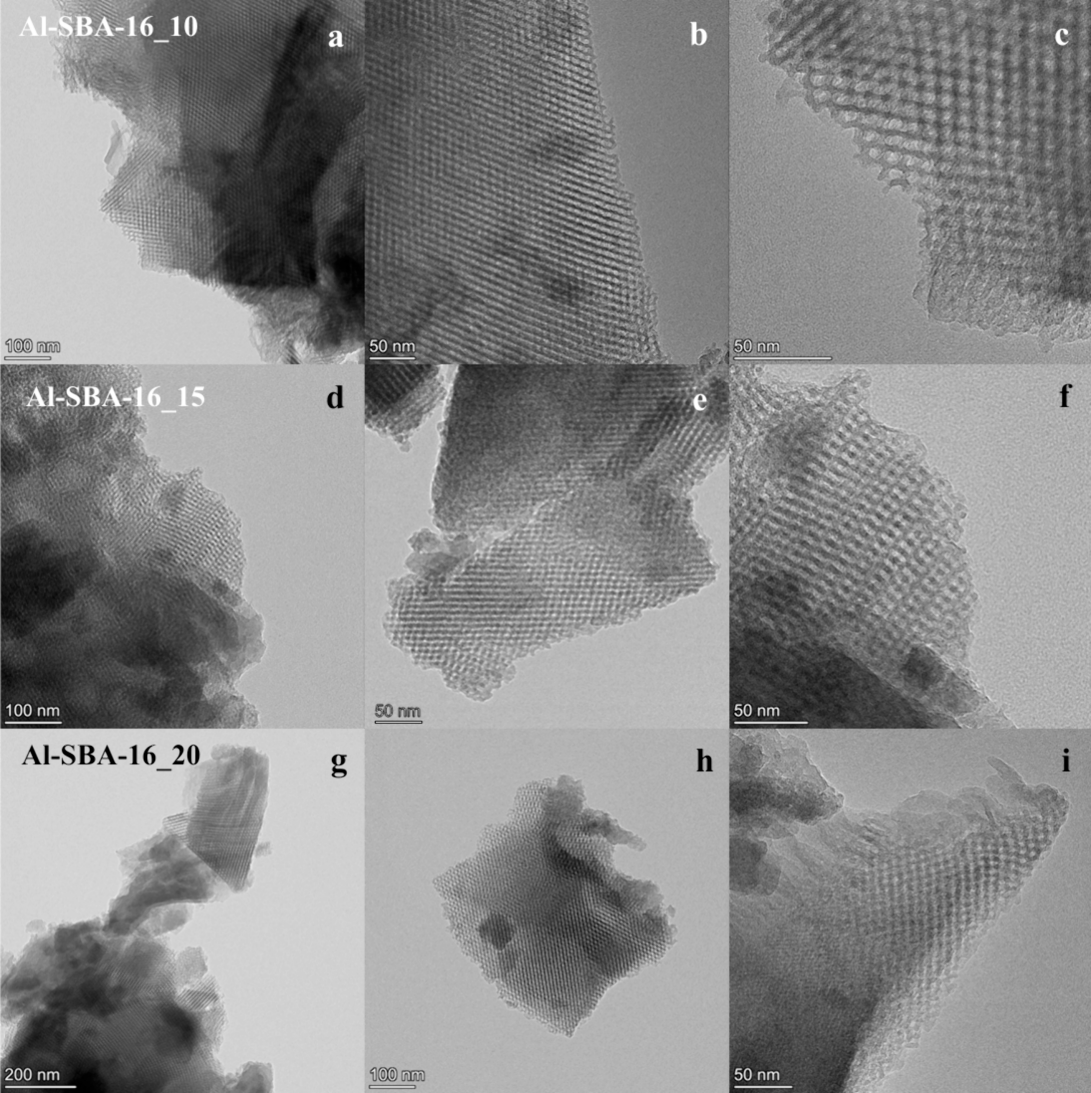
TEM images of the Al-SBA-16 samples with different Si/Al ratios, i.e., 10 (a–c), 15 (d–f), and 20 (g–i), taken at different magnifications.

EDX elemental mapping (Figure 3) revealed a homogeneous distribution of Al and Si across all aluminosilicate samples with no evident phase segregation. However, quantitative EDX analysis performed at different locations showed compositional variations in the Si/Al ratio (Table S1). These discrepancies became more pronounced with increasing aluminum content (i.e., decreasing the Si/Al ratio), suggesting that Al excess tends to segregate as Al_2_O_3_ rather than being fully incorporated into the aluminosilicate framework. This finding is particularly evident for Al-SBA-16_10, which also shows the lowest surface area (Table 1) among the samples, as previously mentioned, further suggesting a segregation of amorphous Al_2_O_3_. For the samples with lower aluminum content (Al-SBA-16_15 and Al-SBA-16_20), the EDX results were in good agreement with those from ICP-OES, confirming the reliability of the EDX technique as a semiquantitative method for aluminosilicates. For Al-SBA-16_10, there is a more evident discrepancy between the results of elemental quantification by ICP-OES and EDX. However, considering that the former result falls within the SD of the latter, and taking into account the intrinsic limitation of EDX as a semiquantitative technique, the two results can be considered in agreement.

**Figure 3:**
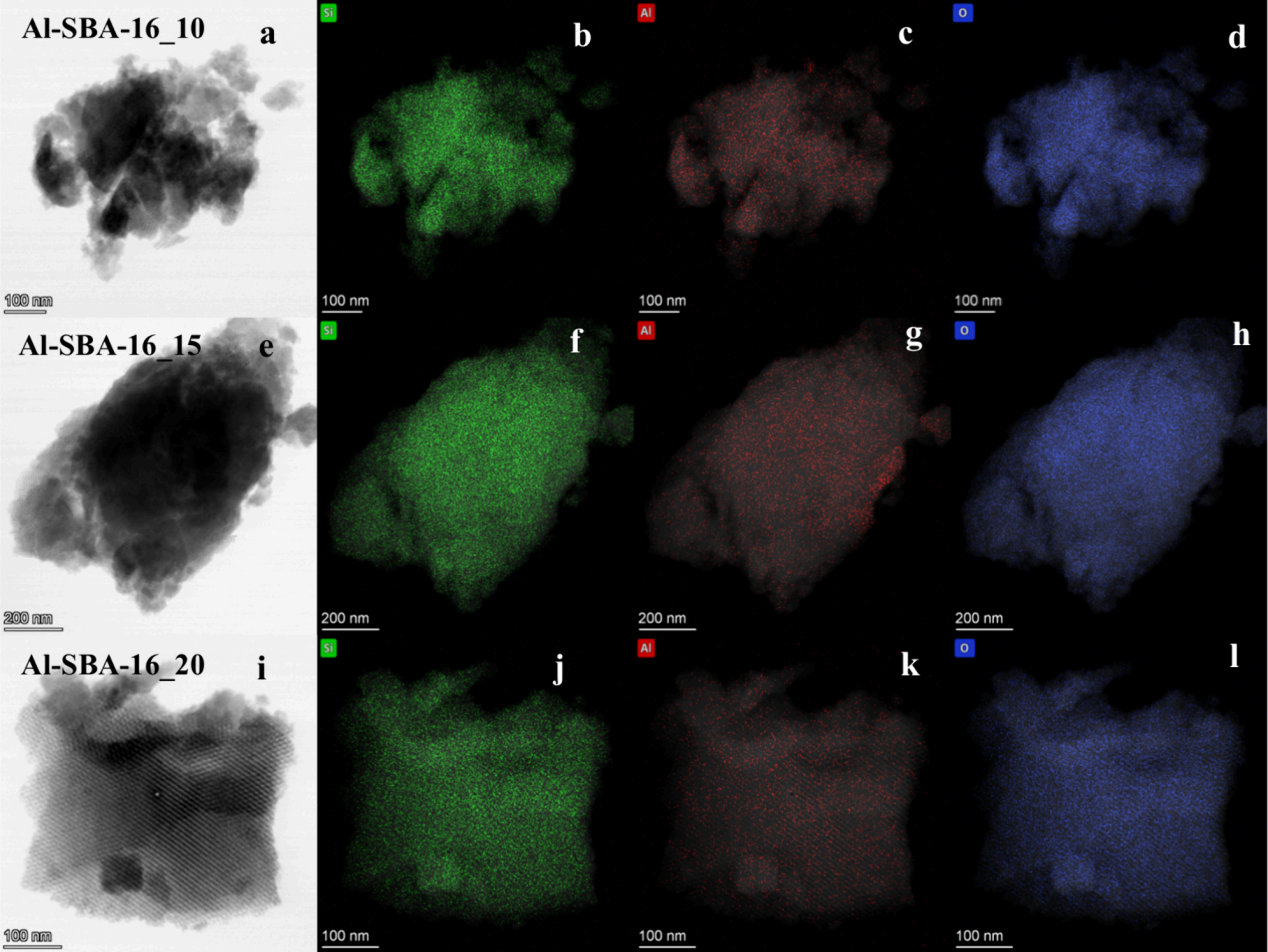
EDX elemental (Si, Al, and O) mapping images of the Al-SBA-16 samples with different Si/Al ratios, i.e., 10 (a–d), 15 (e–h), and 20 (i–l).

TGA was carried out to evaluate the weight loss associated with surface −OH groups (Figure S2b). All three samples feature similar thermogram profiles but with significant differences in total weight loss. Al-SBA-16_10 showed distinct behavior compared with Al-SBA-16_15 and Al-SBA-16_20, which exhibited nearly identical curves. An initial sharp weight loss between 25 and 120 °C was attributed to the desorption of physisorbed water. This weight loss was lower for Al-SBA-16_10 (4.1%) than for Al-SBA-16_15 (5.8%) and Al-SBA-16_20 (6.0%), consistent with its lower surface area (313 vs 381–421 m^2^/g), which reduces water adsorption capacity. Above 120 °C, a gradual weight decrease was observed up to 900 °C, with an inflection point at 500–550 °C, corresponding to the removal of surface −OH groups. Again, Al-SBA-16_10 exhibited a smaller weight loss (2.2%) compared with Al-SBA-16_15 (3.5%) and Al-SBA-16_20 (3.4%), reflecting its lower surface area and thus reduced amount of −OH sites, possibly due to the aforementioned Al_2_O_3_ segregation.

The mesostructured aluminosilicates were tested as methanol dehydration catalysts in a physical mixture with a Cu-based redox catalyst (CZA) for the one-pot conversion of CO_2_ to DME. As shown in Figure 4a, all samples exhibited comparable CO_2_ conversion (5.3–5.7%) and CO selectivity (42.8–43.9%), reflecting the use of the same amount of CZA (50 mg), which governs CO_2_ hydrogenation. In contrast, significant differences were observed in methanol and DME selectivity.

**Figure 4:**
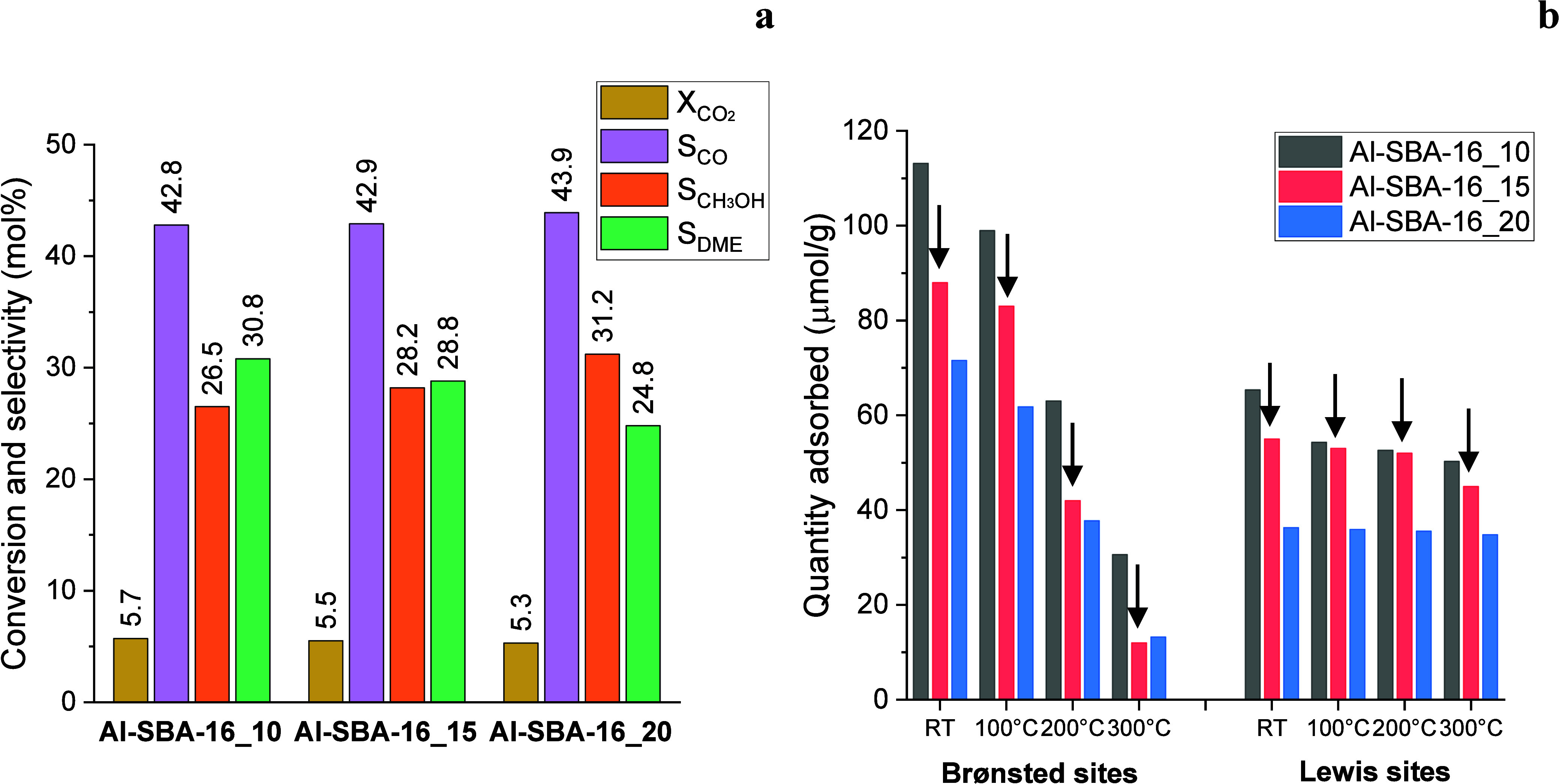
Mean CO_2_ conversion $\left({X_{\mathrm{CO}}}_{2}\right)$ and selectivity to CO (*S*_CO_), methanol (*S*_CH3OH_), and DME (*S*_DME_) obtained from catalytic tests on CZA + Al-SBA-16 physical mixtures. Reaction conditions: time on stream: 24 h; temperature: 250 °C; pressure: 3.0 MPa; GHSV: 48,000 Ncm^3^ g_cat_^–1^ h ^–1^. Weight ratio CZA:Al-SBA-16 = 1:4 (50:200 mg) (a). Quantitative analysis of acid sites determined by pyridine adsorption FT-IR (Py-FT-IR) at increasing temperatures (RT, 100, 200, and 300 °C) on the Al-SBA-16 samples with different Si/Al ratios (i.e., 10, 15, and 20) (b).

DME selectivity increased with decreasing Si/Al ratio (i.e., increasing Al content), consistent with the higher number of acid sites expected at lower Si/Al ratios. Specifically, S_DME_ values were 30.8% for Al-SBA-16_10, 28.8% for Al-SBA-16_15, and 24.8% for Al-SBA-16_20. As expected, S_CH3OH_ followed the opposite trend, ranging from 26.5% (Al-SBA-16_10) to 31.2% (Al-SBA-16_20). However, the improvement in DME selectivity was modest compared to the increase in aluminum content. For example, reducing the Si/Al ratio from 20 to 15 (a 33% increase in Al) resulted in only a 16% relative increase in DME selectivity (24.8 → 28.8%). A further reduction from Si/Al = 15 to 10 (50% more Al) led to just a 7% relative increase (28.8 → 30.8%). These results suggest that while higher aluminum content enhances methanol dehydration, the gain in DME selectivity does not scale proportionally, likely due to limitations in aluminum incorporation into the framework and acid site effectiveness. The catalytic data of Al-SBA-16 samples were compared with those of a mesostructured γ-Al_2_O_3_ sample tested in the same conditions (Figure S3). As expected from the previously obtained results,^47^ the comparison points out distinctly superior performances for the aluminosilicate samples. However, all catalysts showed a gradual decrease in terms of DME selectivity over time (Figure S4), presumably attributable to a partial water poisoning of the acid sites, particularly evident in the first 7.5 h of reaction, after which the catalysts become more stable. Particularly, the deactivation becomes more important as the Si/Al ratio increases. Al-SBA-16_10 showed a −9.3% drop in *S*_DME_ (relative decrease of −24.9%), Al-SBA-16_15 presented a −9.8% decrease (relative decrease of −27.6%), and Al-SBA-16_20 featured a −11.3% drop (relative decrease of −34.6%).

To rationalize the catalytic trends, the acid properties of the aluminosilicates were investigated by NH_3_-TPD and Py-FT-IR analyses. NH_3_-TPD measurements were carried out to estimate the total acidity of the catalysts. All the samples exhibited a very broad desorption profile with a high-temperature tail (Figure S5), indicating the existence of acid sites of different strength, which can be roughly classified as a function of the ammonia desorption temperature as weak (100–250 °C), medium (250–350 °C), and strong (>350 °C).^67,68^ Accordingly, the presence of a well-defined desorption peak at around 200–220 °C indicated that all the samples are characterized by a high percentage of weak acid sites, which can be mainly ascribed to surface hydroxyl groups.^68^ At higher temperatures, the ammonia desorption can be attributed to stronger acid sites (medium and strong Bro̷nsted sites, as well as Lewis sites),^69^ although the lack of clear shoulders in the TPD profiles made their assignment complicated. As expected, the total number of acid sites increased with increasing Al content, with values of 590, 638, and 757 μmol/g calculated for Al-SBA-16_20, Al-SBA-16_15, and Al-SBA-16_10, respectively.

A more detailed investigation of the acid properties was conducted using Py-FT-IR, which enabled quantification of Bro̷nsted and Lewis acid sites (Figure 4b, Table 2, and Figure S6). Measurements were performed between 25 and 300 °C to also assess acid strength. As expected, all samples exhibited both Bro̷nsted and Lewis sites. In agreement with the NH_3_-TPD results, the total number of acid sites followed the order Al-SBA-16_10 > Al-SBA-16_15 > Al-SBA-16_20, consistent with the increasing aluminum content. Notably, the total site number increased by 32% between Al-SBA-16_20 and Al-SBA-16_15, in line with the 33% increase in the aluminum content. For Bro̷nsted sites, this increase (22%) correlated reasonably well with the 16% relative gain in DME selectivity, reinforcing their role as the main active sites for methanol dehydration.

**Table 2:** Quantitative Analysis of Acid Sites by FTIR Spectroscopy with Pyridine as a Probe Molecule^a^

	Al-SBA-16_10				Al-SBA-16_15				Al-SBA-16_20			
	B	L	Tot	B/L	B	L	Tot	B/L	B	L	Tot	B/L
RT	113 (+28%)	65 (+18%)	178 (+24%)	1.7	88 (+22%)	55 (+53%)	143 (+32%)	1.6	72	36	108	2.0
100 °C	99 (+19%)	54 (+2%)	153 (+13%)	1.8	83(+34%)	53 (+47%)	136 (+39%)	1.6	62	36	98	1.7
200 °C	63 (+50%)	53 (+2%)	116 (+23%)	1.2	42 (+11%)	52 (+44%)	94 (+27%)	0.8	38	36	74	1.1
300 °C	31 (+158%)	50 (+11%)	81 (+42%)	0.6	12(−8%)	45 (+29%)	57 (+19%)	0.3	13	35	48	0.4
residual	27%	77%			14%	81%			18%	97%		

a The amount of total acid sites (Tot), Bro̷nsted sites (*B*), and Lewis sites (*L*) is given as μmol/g with the percentage variation indicated between round brackets. The percentage variation reported for Al-SBA-16_15 is in comparison with Al-SBA-16_20; the one reported for Al-SBA-16_10 is in comparison with Al-SBA-16_15.

In contrast, the comparison between Al-SBA-16_15 and Al-SBA-16_10 revealed that a 50% increase in the aluminum content produced only a 24% increase in total acid sites, suggesting that a fraction of Al does not contribute to active site formation, likely forming inactive or bulk Al species. This hypothesis is supported by the smaller surface area of Al-SBA-16_10. For Bro̷nsted sites specifically, the increase from Al-SBA-16_15 to Al-SBA-16_10 was 28%, yet the corresponding improvement in DME selectivity was only 7%; the trend of DME selectivity as a function of the number of Bro̷nsted sites was graphically represented in Figure S7. This discrepancy can be explained by differences in the acid strength: Py-FT-IR desorption profiles showed that Bro̷nsted sites (arrows in Figure 4b for Al-SBA-16_15 as an example) were generally weaker than Lewis sites (arrows in Figure 4b for Al-SBA-16_15 as an example), with progressive pyridine loss upon heating. Furthermore, it can be observed that Lewis sites become stronger with a decrease in the Al content of the aluminosilicate sample. Comparing the amount of Lewis sites of Al-SBA-16_10 at RT and 300 °C, it can indeed be observed that it goes from 65 to 50 μmol/g, corresponding to 77% of residual sites with pyridine still adsorbed at 300 °C (Table 2). Decreasing the Al content, this percentage reaches 81% for Al-SBA-16_15 and 97% for Al-SBA-16_20. An opposite behavior, despite being less evident, can be observed for the Bro̷nsted acid sites, which seems to decrease their strength, decreasing the Al content of the sample. Indeed, a significant difference can be observed between Al-SBA-16_10, with 27% of remaining Bro̷nsted sites at 300 °C, and the other two catalysts, with a lower percentage (14% for Al-SBA-16_15 and 18% for Al-SBA-16_20). This finding might justify the relatively low increase (7%) in catalytic activity observed between Al-SBA-16_10 and Al-SBA-16_15, compared with the 28% increase in the number of Bro̷nsted sites. Strong Bro̷nsted sites, indeed, are less active toward methanol dehydration compared with low- and medium-strength sites. Furthermore, this result correlates well with the S_DME_ performance loss observed over time. Indeed, as strong Lewis acid sites are more prone to deactivation due to water poisoning than weak and medium sites, it can be assumed that the more pronounced deactivation over time of the aluminosilicates with a higher Si/Al ratio is due to the presence of stronger Lewis sites on their surface. At room temperature, Bro̷nsted sites dominated (*B*/*L* = 1.6–2.0), but the ratio approached unity at 200 °C (*B*/*L* = 0.8–1.2) and fell below 0.6 at 300 °C due to Bro̷nsted site desorption, leaving Lewis sites predominant at higher temperatures.

The ^27^Al single-quantum (1Q) MAS NMR spectra (Figure 5a) exhibit resonances in three regions: ca. −10 to 10, 20–40, and 45–70 ppm, typical of six-, five-, and four-coordinated Al species, respectively. Because ^27^Al is a quadrupolar nucleus (*I* = 5/2), second-order quadrupolar broadening limits spectral resolution, so further insight into local environments relies on triple-quantum (3Q) MAS NMR (Figures 6 and[Fig fig7]).

**Figure 5:**
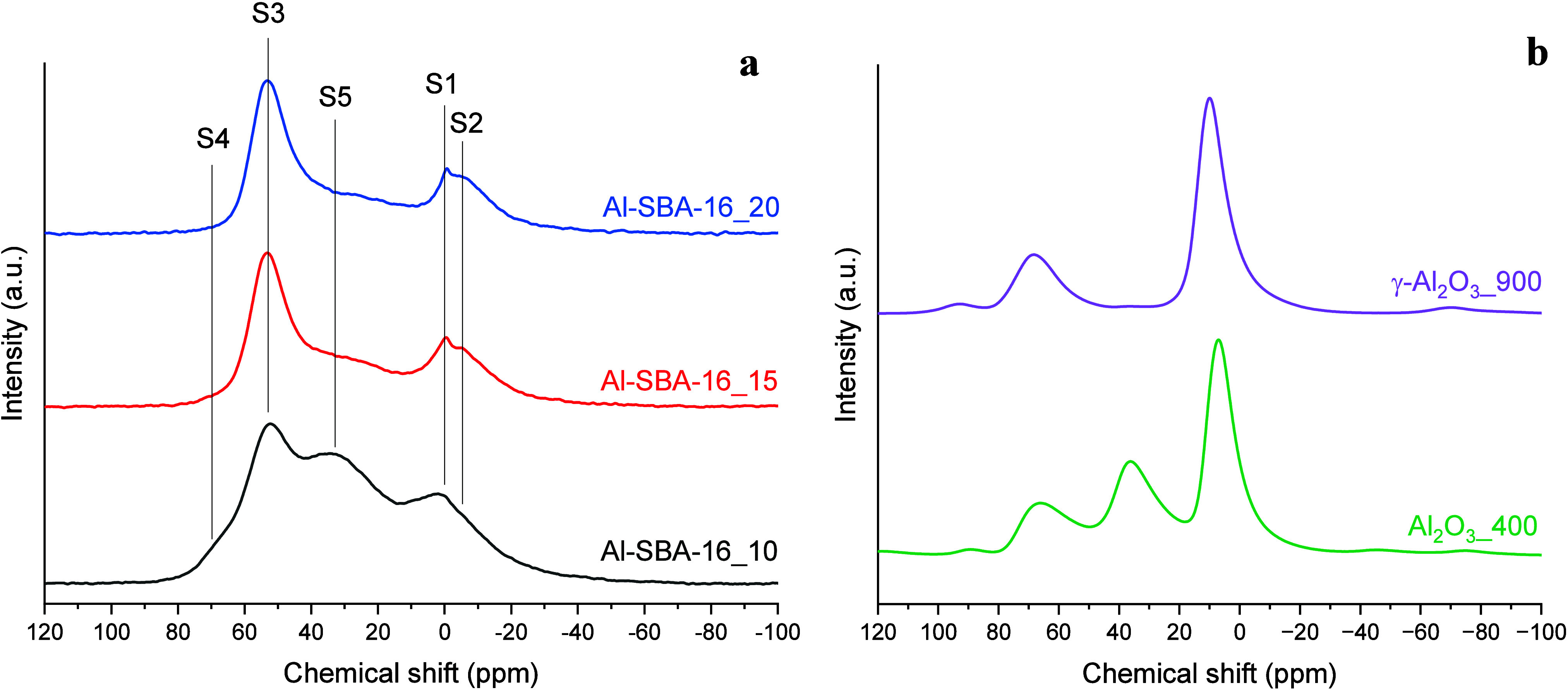
^27^Al single-quantum MAS NMR spectra of the Al-SBA-16 samples with different Si/Al ratios (i.e., 10, 15, and 20) (a); ^27^Al single-quantum MAS NMR spectra of Al_2_O_3_ samples (b).

**Figure 6:**
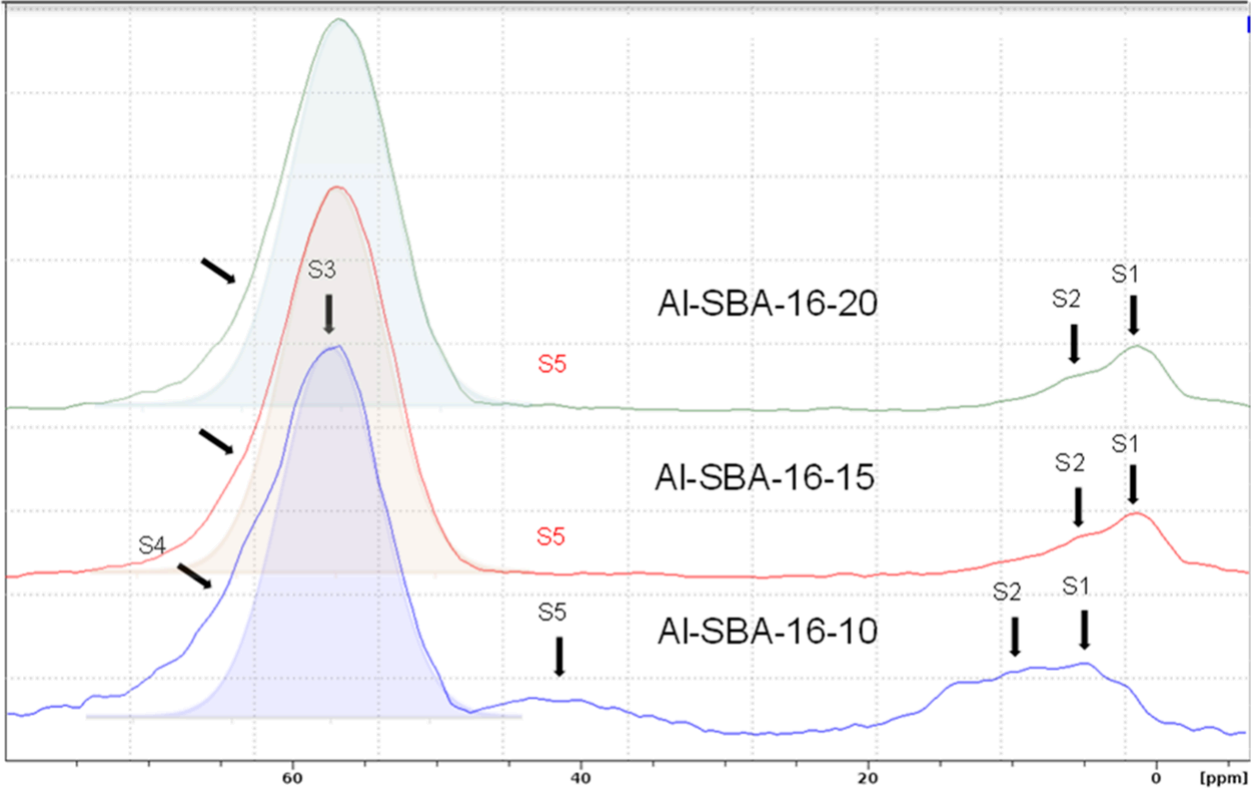
F1 projections of ^27^Al 3Q MAS NMR spectra of the Al-SBA-16 samples with different Si/Al ratios (i.e., 10, 15, and 20).

**Figure 7:**
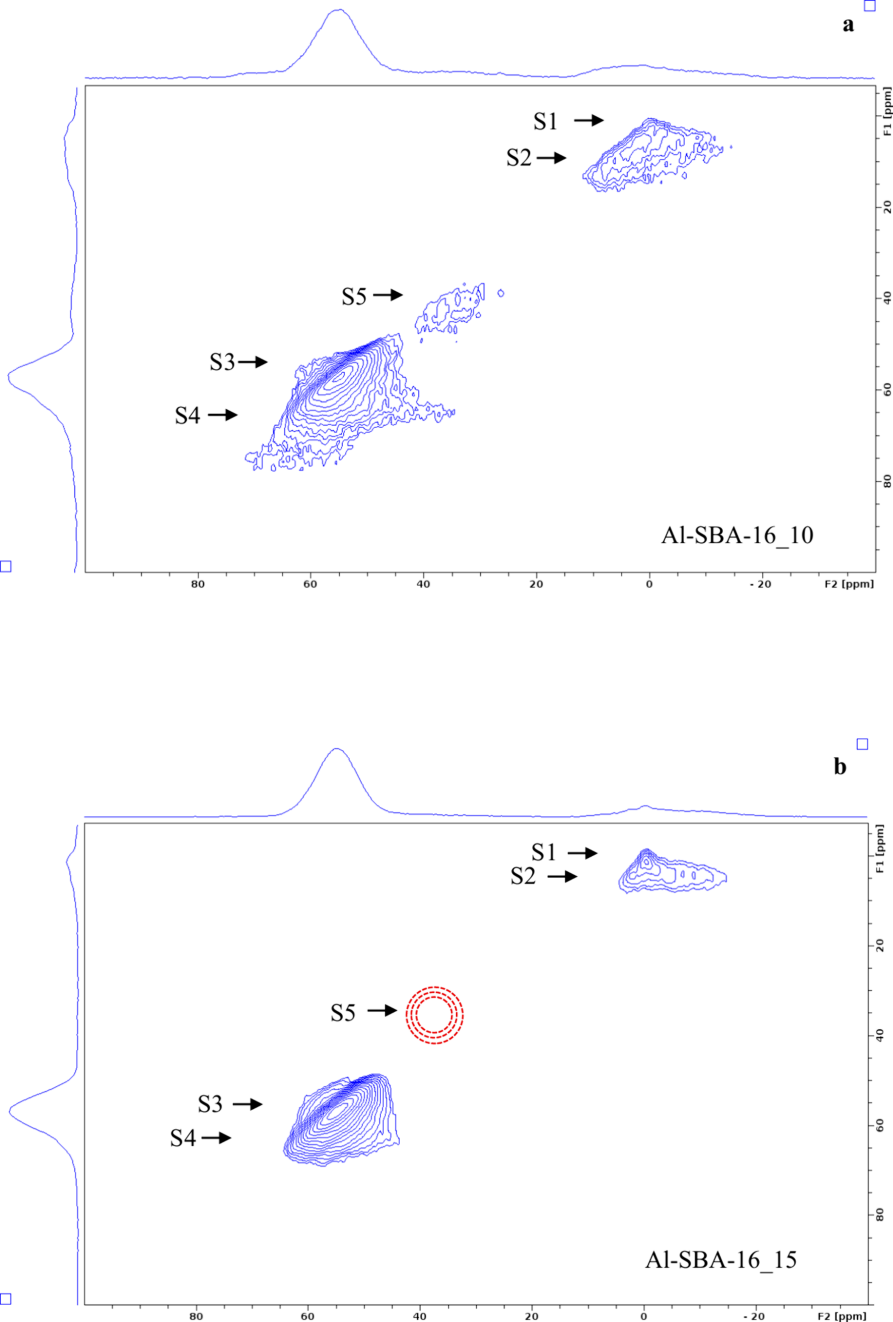

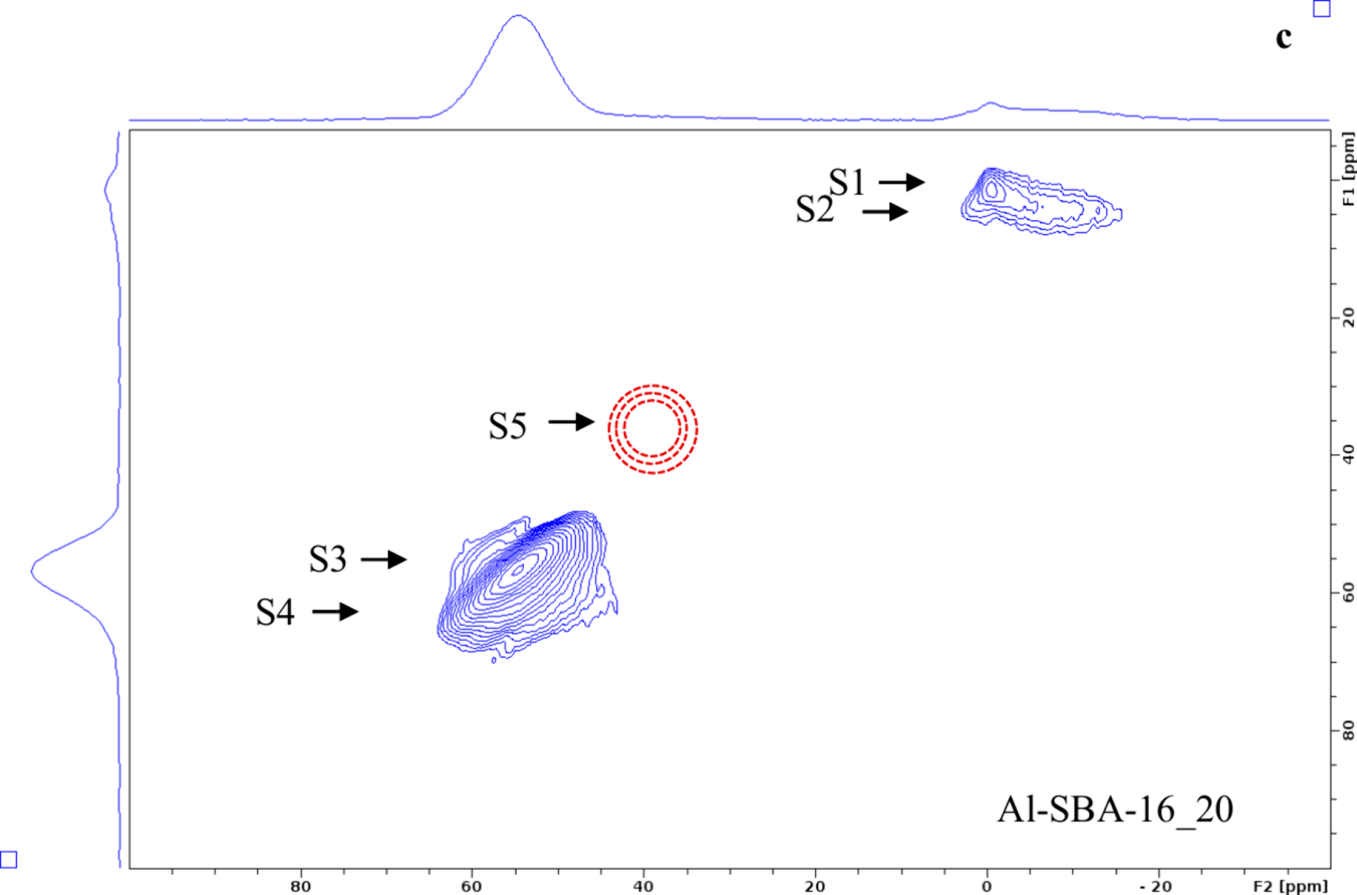
^27^Al 3Q MAS NMR spectra of the Al-SBA-16 samples with different Si/Al ratios, i.e., 10 (a), 15 (b), and 20 (c).

In the 3Q spectra, a dominant resonance (S3) at 53 ppm in the F1 isotropic projections (Figure 6) and in the 2D spectra (Figure 7) is assigned to four-coordinated Al in framework Al–(O–Si)_4_ tetrahedra.^70–73^ Framework tetrahedral Al, when charge-balanced by H^+^, gives rise to Bro̷nsted OH groups, whereas extra-framework tetrahedral Al in alumina-like domains does not, an essential distinction for interpreting the contribution of tetrahedral Al to catalytic activity. With increasing Al content (from Al-SBA-16_20 to Al-SBA-16_10), a second tetrahedral-site resonance (S4) emerges at a higher frequency (ca. 69 ppm), which was attributed to tetracoordinated Al in extra-framework amorphous γ-Al_2_O_3_-like domains; considered together with the strong 1455 cm^–1^ Py-FT-IR band, this is associated with Lewis acidity.^74,75^ This 69 ppm signal is especially prominent in Al-SBA-16_10 and matches the resonance observed for an amorphous alumina reference synthesized via a similar EISA approach (Al_2_O_3__400, Figures 5b and S1b), indicating that at high Al loadings part of the Al segregates as amorphous Al_2_O_3_, consistent with the local compositional inhomogeneities from EDX.

The broad S5 resonance at 20–40 ppm in the 1Q MAS spectra is faint in the lower-Al samples. Because 3Q excitation and conversion efficiencies depend on RF/quadrupolar coupling frequency, very large quadrupolar couplings can reduce the apparent S5 intensity in 3Q under our conditions. S5 can be assigned to five-coordinated^76,77^ (or highly distorted tetrahedral) Al species located outside the framework or on the surface, possibly coordinated to hydroxyl groups. Its strong intensity in both Al-SBA-16_10 and the amorphous Al_2_O_3_ reference indicates that S5 mainly originates from amorphous Al_2_O_3_, confirming its significant presence in Al-SBA-16_10.

In the low-frequency region, the 1Q MAS spectra display a narrow resonance at −1 ppm (S1; hardly discernible in Al-SBA-16_10) and a broader peak at ∼−6 ppm (S2), both corresponding to octahedral Al species,^70–73^ as confirmed by 3Q MAS. The −1 ppm signal is characteristic of highly symmetric six-coordinated Al, likely small amounts of hydrated Al ions in the pores, consistent with WA-XRD showing no crystalline phases (Figure S1a). The −6 ppm resonance is attributed to octahedral Al in amorphous Al_2_O_3_; its intensity increases with Al content and is particularly pronounced in Al-SBA-16_10, indicating segregation of Al as amorphous Al_2_O_3_ rather than incorporation into the silica framework.

These octahedral Al atoms, the only Al species present in α-Al_2_O_3_, when coordinatively unsaturated, only show a weak Lewis acidity,^78,79^ thus being catalytically inert in methanol dehydration,^40^ explaining why the increase in total acid sites from Al-SBA-16_15 to Al-SBA-16_10 (∼24%) is much lower than the increase in Al content (50%), and why the improvement in DME selectivity is modest (∼7%).

Because precise quantification of Al species from the ^27^Al 1Q MAS NMR spectra is hindered by second-order quadrupolar effects and peak overlap, a semiquantitative analysis was performed by integrating the signal areas within three regions: −30 to 10 ppm (six-coordinated Al), 10–40 ppm (five-coordinated Al), and 40–80 ppm (four-coordinated Al) (Table 3). Within the limits of this approach, the ratio of four- to six-coordinated Al remained consistently ∼1.5 across all samples, indicating that the overall Al content does not strongly affect this balance. The most significant variation was observed in the fraction of five-coordinated Al, which decreased from 35% in Al-SBA-16_10 to 22% in Al-SBA-16_20. This trend suggests that higher Al loading favors the formation of five-coordinated species.

**Table 3:** Semiquantitative Distribution of Four-, Five-, and Six-Coordinated Al Species as Estimated from ^27^Al Single-Quantum MAS NMR Spectra and Results of the Deconvolution of the ^29^Si MAS NMR Spectra of the Al-SBA-16 Samples with Different Si/Al Ratios (i.e., 10, 15, and 20)

sample	Al 6-coord (−30–10 ppm) (%)	Al 5-coord (10–40 ppm) (%)	Al 4-coord (40–80 ppm) (%)	Si Q4 (%)	Si Q3 (%)	Si Q2 (%)
Al-SBA-16_10	26	35	39	69	27	4
Al-SBA-16_15	30	24	45	73	25	2
Al-SBA-16_20	31	22	47	63	32	5

The ^29^Si MAS spectra (Figure 8a) display a broad Q^4^ envelope centered at −109 ppm with shoulders at −100 and −91 ppm, assigned to Q^3^ and Q^2^, respectively, consistent with amorphous silica–aluminosilicate networks.^70,80,81^ Deconvolution yields 63% Q^4^, 32% Q^3^, and ∼5% Q^2^ (Table 3), invariant within ±10% across samples, indicating a highly condensed silicate framework. The slight increase in the Q^3^ + Q^2^ fraction with Al loading suggests additional defect/silanol sites and some Si–O–Al formation. This is corroborated by ^1^H–^29^Si CP-MAS (Figure 8b), which selectively enhances the −100 and −91 ppm signals, confirming the proximity to protons (silanols/Al–OH). Notably, the Q^4^ band shifts slightly toward high frequency as Al increases, consistent with partial framework substitution (Q^4^(1Al), Si–O–Al); however, the limited magnitude of this shift, together with ^27^Al evidence for extra-framework Al_2_O_3_ at high loading, indicates that framework Al insertion grows only modestly, while extra-framework alumina becomes significant. This reconciles the moderate rise in Bro̷nsted site number from Py-FT-IR with the only modest gain in DME selectivity at the lowest Si/Al ratio.

**Figure 8:**
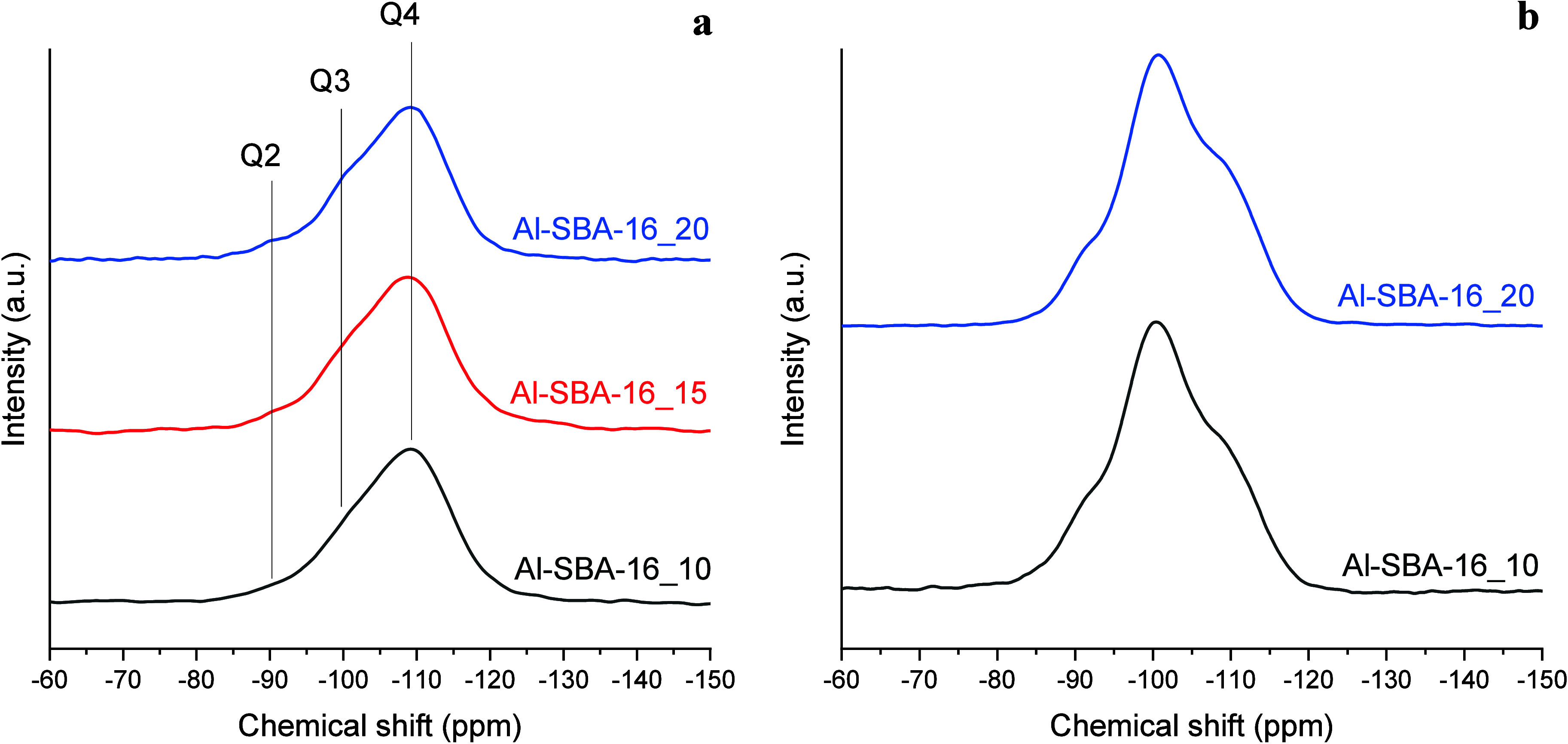
^29^Si MAS NMR spectra of the Al-SBA-16 samples with different Si/Al ratios (i.e., 10, 15, and 20) (a) and ^1^H- ^29^Si- CP-MAS spectra of selected Al-SBA-16 samples (b).

## Conclusions

In this study, mesostructured Al-SBA-16 catalysts with Si/Al ratios of 10, 15, and 20 were evaluated for methanol dehydration in a one-pot CO_2_-to-DME conversion. Structural, textural, compositional, and spectroscopic analyses clarified how Al content, coordination, and distribution control acidity and catalytic behavior.

DME selectivity increased with decreasing Si/Al ratio but less than expected from the nominal Al increment. Pyridine-FTIR spectroscopy confirmed that higher Al loadings generated more acid sites with nearly constant Bro̷nsted/Lewis ratios. The correlation between the Bro̷nsted site number and DME selectivity was evident from Si/Al = 20 to 15 but weakened at higher Al contents, indicating diminishing catalytic gains.

Solid-state ^27^Al NMR revealed the origin of this discrepancy: while tetrahedral framework Al generates Bro̷nsted sites, higher Al loadings favor the formation of five- and six-coordinated species in extra-framework amorphous Al_2_O_3_, which are catalytically inactive. Consistently, ^29^Si MAS/CP-MAS NMR showed a slight increase in Q^3^/Q^2^ contributions and only a subtle shift of the Q^4^ envelope with Al, indicating modest framework Al insertion alongside the growth of extra-framework alumina. The Al-SBA-16_10 sample, in particular, exhibited significant alumina segregation, which is consistent with EDX inhomogeneities and the lower surface area from N_2_ physisorption.

Overall, catalytic performance depends more on Al incorporation than on total Al content with a practical upper limit beyond which inactive alumina phases form. Future work should therefore refine sol–gel-based synthesis strategies (in both their conventional and EISA approaches), e.g., using alternative precursors to AlCl_3_ (like Al­(NO_3_)_3_ and different Al alkoxides) or controlled gelation/humidity, to maximize framework Al incorporation and Bro̷nsted acidity, while managing acid strength, thereby improving methanol dehydration performance in CO_2_-to-DME conversion.

## Supplementary Material



## Notes

Wide-angle XRD data of Al-SBA-16 and Al_2_O_3_ samples (Figure S1), EDX analyses results on Al-SBA-16 samples (Table S1), BJH and TGA curves of Al-SBA-16 samples (Figure S2), catalytic tests of Al-SBA-16 samples compared with γ-Al_2_O_3_ (Figure S3), over time stability of the catalytic performance of Al-SBA-16 samples (Figure S4), NH_3_-TPD desorption profiles of Al-SBA-16 samples (Figure S5), pyridine-FTIR spectra of Al-SBA-16 samples (Figure S6), and DME selectivity trend as a function of the number of Bro̷nsted acid sites for Al-SBA-16 samples (Figure S7) (PDF)
