# Supplementary material for: Beyond Aluminum Loading: How Aluminum Coordination Controls Acidity and Catalytic Performance of Al-SBA-16 in CO2‑to-DME Conversion
Source: ACS Appl Mater Interfaces. 2025 Dec 29;18(1):1242–55. doi: 10.1021/acsami.5c18904 (PMC12781109; doi:10.1021/acsami.5c18904)
Supplement: Supplementary file 1 [file am5c18904_si_001.pdf]

Supporting information

Beyond Aluminum Loading: How Aluminum Coordination Controls Acidity and Catalytic Performance of Al-SBA-16 in CO_2-to-DME Conversion

Fausto Secci,[1-2] Valentina Mameli,[1-2] Patrícia A. Russo,[3] Paula Soares-Santos,[4] Luciano Atzori,[1-2] Mauro Mureddu,[5] Nicola Pinna,[3] João Rocha,[4*] Carla Cannas[1-2*]

[1] Department of Chemical and Geological Sciences, University of Cagliari, S.S.554 bivio per Sestu, 09042 Monserrato, CA, Italy

[2] Consorzio Interuniversitario Nazionale per la Scienza e Tecnologia dei Materiali (INSTM), Via Giuseppe Giusti 9, 50121 Firenze, FI, Italy

[3] Department of Chemistry and The Center for the Science of Materials Berlin, Humboldt-Universität zu Berlin, Berlin, 12489 Germany

[4] CICECO – Aveiro Institute of Materials, Department of Chemistry, University of Aveiro, Aveiro 3810-193, Portugal

[5] Sotacarbo S.p.A., Grande Miniera di Serbariu, 09013 Carbonia, SU, Italy

*Corresponding authors

e-mail: ccannas@unica.it, rocha@ua.pt

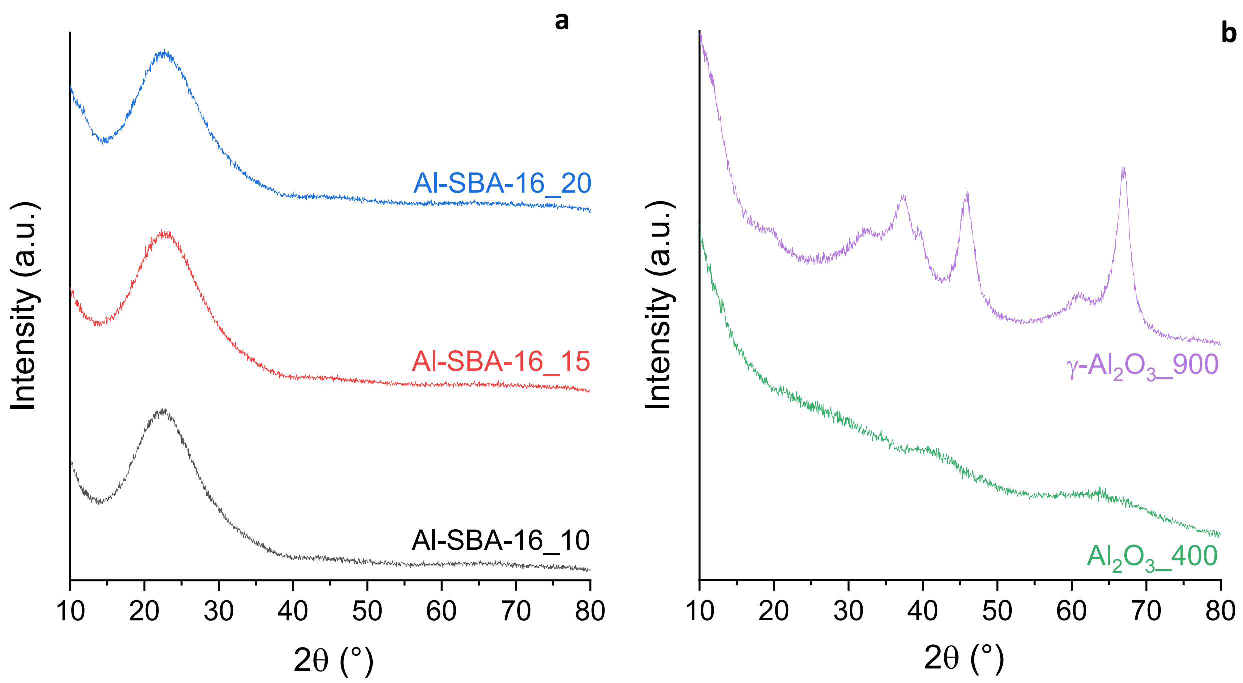

Figure S1 SA-XRD patterns of the Al-SBA-16 samples with different Si/Al ratios (i.e. 10, 15, and 20) (a), and of the Al_2O_3 samples (b). The patterns depicted in (b) are only reported as a reference and have been published in a previous work. [15]

Table S1 Atomic percentages of Si and Al determined from EDX spectral analysis on different sites of the Al-SBA-16 samples with different Si/Al ratios (i.e. 10, 15, and 20).

**Al-SBA-16_10**	**Si atomic %**	**Al atomic %**	**Si/Al atomic ratio**	**Mean Si/Al + SD**
**Measure 1**	24.6	7.47	3.29	6.9 (3.6)
**Measure 2**	29.0	3.31	8.76	
**Measure 3**	26.3	5.16	5.10	
**Measure 4**	26.8	5.16	5.19	
**Measure 5**	28.1	2.30	12.22	
**Al-SBA-16_15**				
**Measure 1**	30.2	2.32	13.02	14.1 (1.6)
**Measure 2**	29.6	1.86	15.91	
**Measure 3**	29.3	2.21	13.26	
**Al-SBA-16_20**				
**Measure 1**	29.6	1.69	17.51	17.5 (0.14)
**Measure 2**	28.4	1.64	17.32	
**Measure 3**	29.2	1.66	17.59	

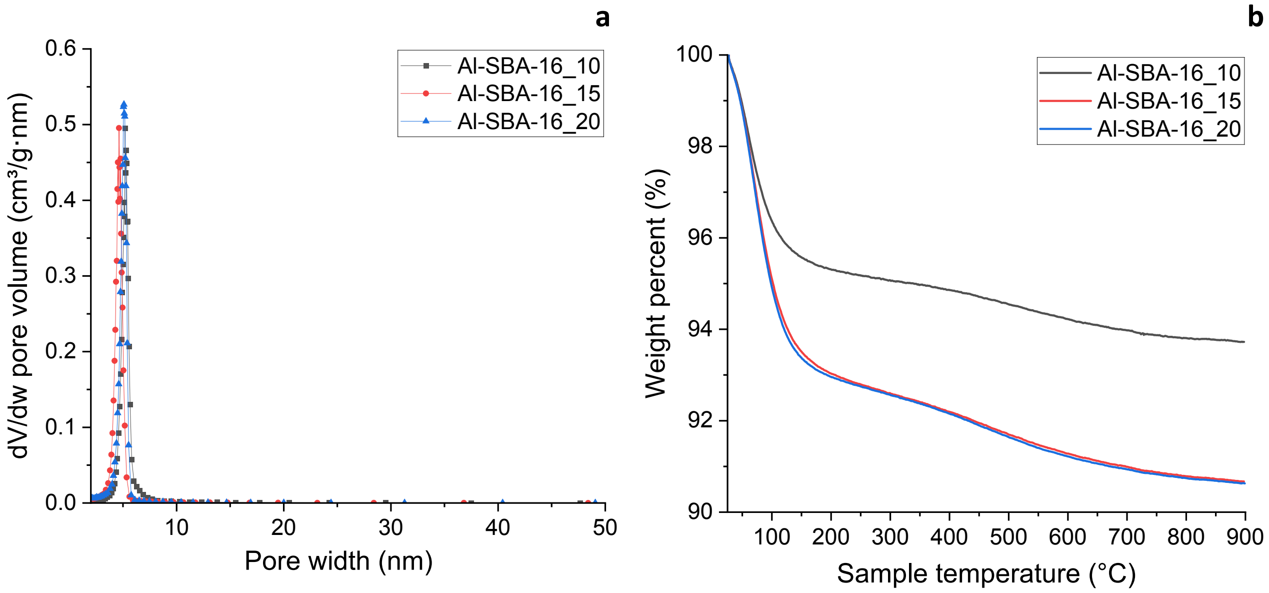

Figure S2 BJH adsorption pore size distribution in the range 2-50 nm (a) and thermogravimetric analysis (b) of the Al-SBA-16 samples with different Si/Al ratios (i.e. 10, 15, and 20).

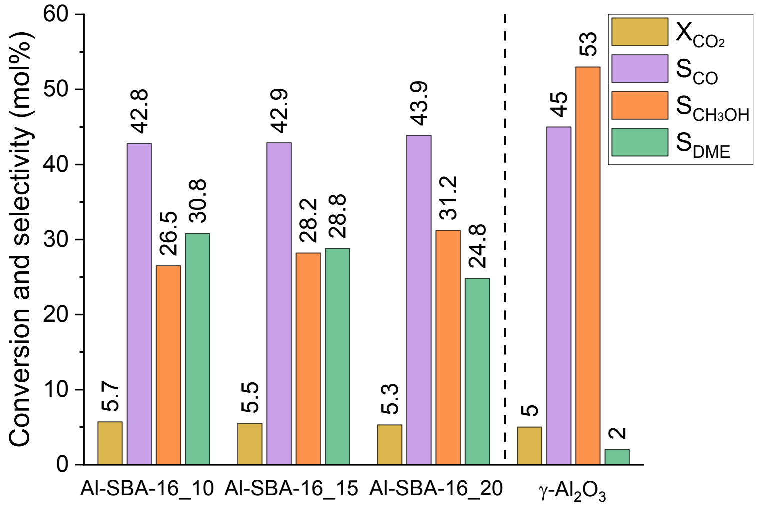

Figure S3 Mean CO_2 conversion (X_{CO2}) and selectivity to CO (S_{CO}), methanol (S_{CH3OH}), and DME (S_{DME}) obtained from catalytic tests on CZA + Al-SBA-16 physical mixtures compared with those of a CZA + mesostructured γ-Al_2O_3 physical mixture tested with the same conditions in a previous work. [15]

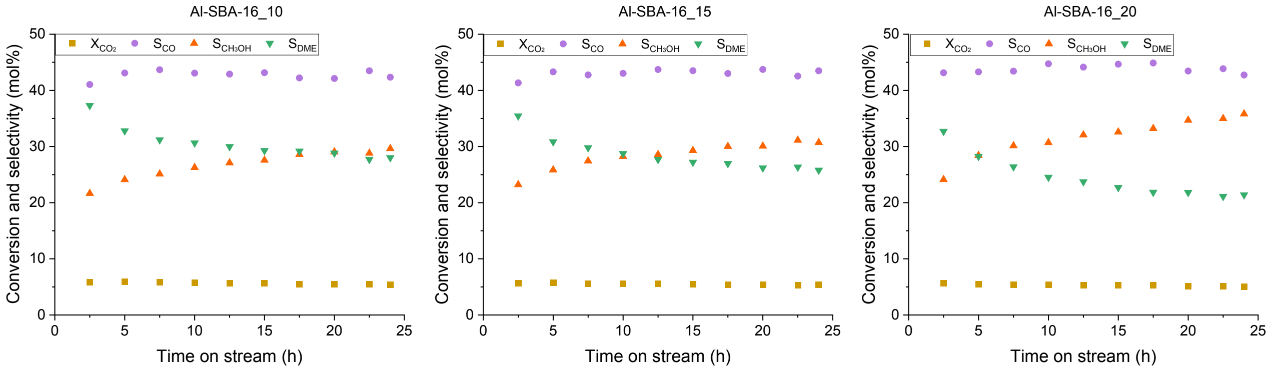

Figure S4 Values of CO_2 conversion (X_{CO2}) and selectivity to CO (S_{CO}), methanol (S_{CH3OH}), and DME (S_{DME}) over time obtained from catalytic tests on CZA + Al-SBA-16 physical mixtures. Reaction conditions: time on stream: 24 h; temperature: 250 °C; pressure: 3.0 MPa; GHSV: 48,000 $Ncm^3\ g_{cat}^{-1}\ h^{-1}$. Weight Ratio CZA:Al-SBA-16 = 1:4 (50 mg : 200 mg).

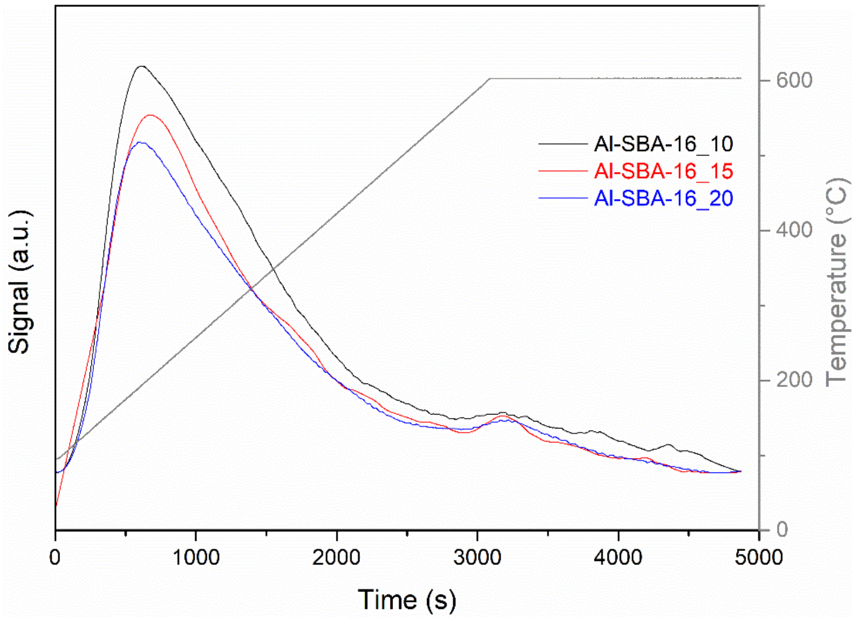

Figure S5 NH3-TPD curves obtained on the Al-SBA-16 samples with different Si/Al ratios, i.e. 10, 15, and 20.

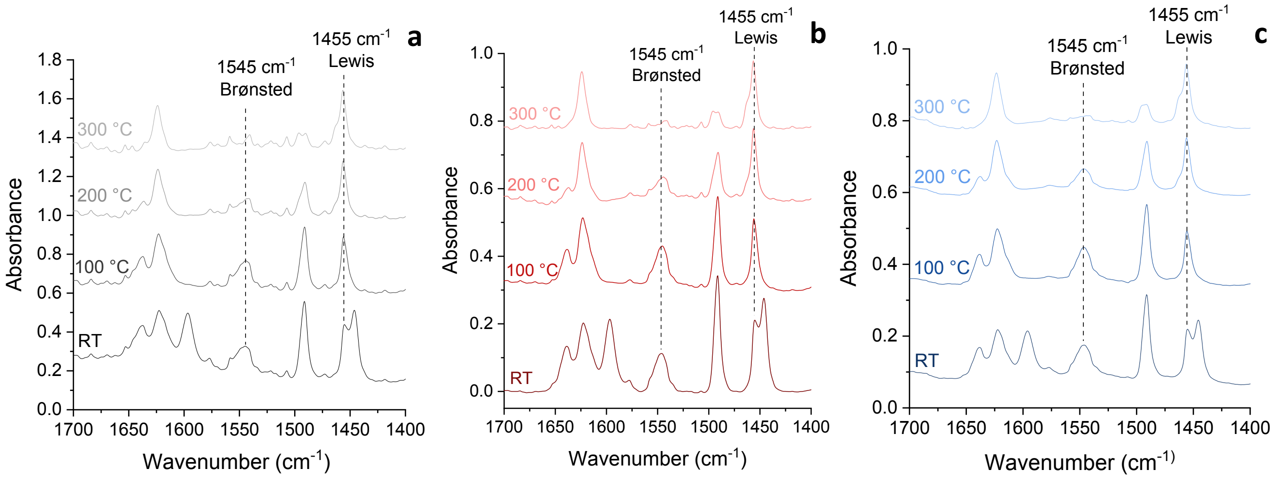

Figure S6 Pyridine-FTIR spectra of the Al-SBA-16 samples with different Si/Al ratios, i.e. 10 (a), 15 (b), and 20 (c).

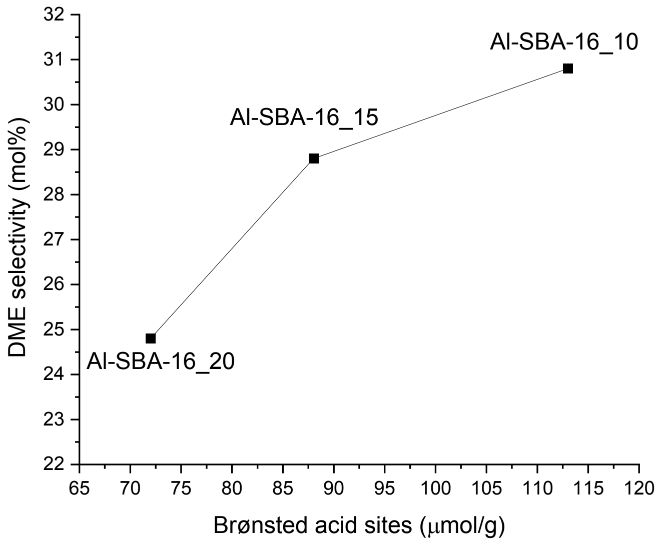

Figure S7 DME selectivity trend as a function of the number of Brønsted acid sites.
